# Insulin‐like growth factor‐II and bioactive proteins containing a part of the E‐domain of pro‐insulin‐like growth factor‐II

**DOI:** 10.1002/biof.1623

**Published:** 2020-02-06

**Authors:** Jaap van Doorn

**Affiliations:** ^1^ Department of Genetics, Section Metabolic Diagnostics University Medical Center Utrecht, Utrecht University Utrecht The Netherlands

**Keywords:** big IGF‐II, bone turn over, E‐domain, glucose homeostasis, hepatitis C‐associated osteosclerosis, IGF‐binding protein, IGF‐II, non‐islet cell tumor hypoglycemia, preptin, pro‐IGF‐II

## Abstract

Insulin‐like growth factor (IGF)‐II is considered to function as an important fetal growth factor, which is structurally and functionally related to IGF‐I and proinsulin. At least in vitro, IGF‐II actions are mediated through the IGF‐I receptor and to a lesser extent the insulin receptor. After birth, the function of IGF‐II is less clear although in adults the serum level of IGF‐II exceeds that of IGF‐I several fold. The IGF‐II gene is maternally imprinted, with exception of the liver and several parts of the brain, where it is expressed from both alleles. The regulation, organization, and translation of the IGF‐II gene is complex, with five different putative promotors leading to a range of noncoding and coding mRNAs. The 180‐amino acid pre‐pro‐IGF‐II translation product can be divided into five domains and include a N‐terminal signal peptide of 24 amino acid residues, the 67 amino acid long mature protein, and an 89 residues extension at the COOH terminus, designated as the E‐domain. After removal of the signal peptide, the processing of pro‐IGF‐II into mature IGF‐II requires various steps including glycosylation of the E‐domain followed by the action of endo‐proteases. Several of these processing intermediates can be found in the human circulation. There is increasing evidence that, besides IGF‐II, several incompletely processed precursor forms of the protein, and even a 34‐amino acid peptide (preptin) derived from the E‐domain of pro‐IGF‐II, exhibit distinct biological activities. This review will focus on the current insights regarding the specific roles of the latter proteins in cancer, glucose homeostasis, and bone physiology. To address this topic clearly in the right context, a concise overview of the biological and biochemical properties of IGF‐II and several relevant aspects of the IGF system will be provided.

**Abbreviations**
ALS: acid labile subunit
CAC: coronary artery calcification
CTGF: connective tissue growth factor
ERK: extracellular signal‐regulated kinase
GH: growth hormone
GHRH: growth hormone‐releasing hormone
GIST: gastrointestinal stromal tumors
GLUT4: glucose transporter 4
GRP: glucose‐regulated protein
HBV‐X: hepatitis B viral protein‐X
HCAO: hepatitis C‐associated osteosclerosis
HEK: human embryonic kidney
HOMA‐IR: homeostatic model assessment insulin resistance
IGF: insulin‐like growth factor
IGFBP: IGF‐binding protein
IGF‐IR: IGF‐I receptor
IR: insulin receptor
LOH: loss of heterozygosity
M6P: mannose‐6‐phosphate
MAPK: mitogen‐activated protein kinase
MM: molecular weight
M6P/IGF‐IIR: mannose‐6‐phosphate/IGF‐II receptor
NICTH: non‐islet cell tumor‐induced hypoglycemia
PAPP‐A2: matrix metallopeptidase pregnancy‐associated plasma protein A2
PC: prohormone convertase
PCSK4: subtilisin/kexin type 4
PKB: protein kinase B
PKC: phosphokinase C
PLC: phospholipase C
SS: somatostatin

## 1 INTRODUCTION

The growth hormone (GH)‐insulin‐like growth factor (IGF)‐I axis is an important hormonal system that is involved in human growth. GH is produced by the anterior pituitary gland in a pulsatile fashion and under the control of the hypothalamic hormones growth hormone‐releasing hormone (GHRH) and somatostatin (SS). GH promotes growth of many tissues, including muscle, cartilage, and bone. Although GH exerts several biological effects intrinsically, most of its actions are mediated by IGF‐I. GH stimulates the expression of IGF‐I after binding to its receptor which is present on many cell types.^1^, ^2^ IGF‐I may exert its proliferative and antiapoptotic effects both in an autocrine, paracrine, or endocrine fashion. The liver is a main contributor to the circulating IGF‐I pool. The GH‐IGF‐I axis is subject to feedback regulation, whereby IGF‐I in particular inhibits the production of both GH and GHRH and stimulates that of SS.^1^, ^2^

In contrast to IGF‐I, the synthesis of IGF‐II is not directly controlled by the action of GH.^3^ As IGF‐I, IGF‐II is synthesized by many tissues, but the liver is the predominant source of IGF‐II in the circulation. IGF‐II is considered to function as an important fetal growth factor which is structurally and functionally related to IGF‐I and (pro) insulin.^4^, ^5^ Postnatally, IGF‐I, instead of IGF‐II, acts as a dominant stimulator of somatic growth and inhibitor of apoptosis. Nevertheless, in the circulation of healthy adults the total concentration of IGF‐II exceeds that of IGF‐I by more than threefold. IGF‐II also serves as a second signal for oncogene‐induced tumorigenesis or overgrowth syndromes such as Beckwith–Wiedemann syndrome.^5^, ^6^, ^7^ Multiple mechanisms have evolved to suppress inappropriate IGF‐II signaling after birth. These consist of genomic imprinting, sequestration by IGF‐binding proteins (IGFBPs), and local removal of IGF‐II from interstitial fluid by the mannose‐6‐phosphate (M6P)/IGF‐II receptor. At least in vitro, IGF‐II actions are mediated through the IGF‐I receptor (IGF‐IR) and the insulin receptor (IR). There is increasing evidence that, besides fully processed mature 7.5 kD IGF‐II, also incompletely processed precursor forms of the protein, and even a 34‐amino acid peptide (preptin) derived from the E‐domain of pro‐IGF‐II, exhibit distinct biological activities. This review will focus on the current insights regarding the possible roles of the latter proteins in cancer, glucose homeostasis, and bone physiology. Before addressing this subject further, a concise overview of relevant biological and biochemical aspects of IGF‐II and the IGF system will be addressed.

## 2 BIOLOGICAL AND BIOCHEMICAL ASPECTS OF IGF‐II

### 2.1 IGF‐binding proteins

**Figure 1:**
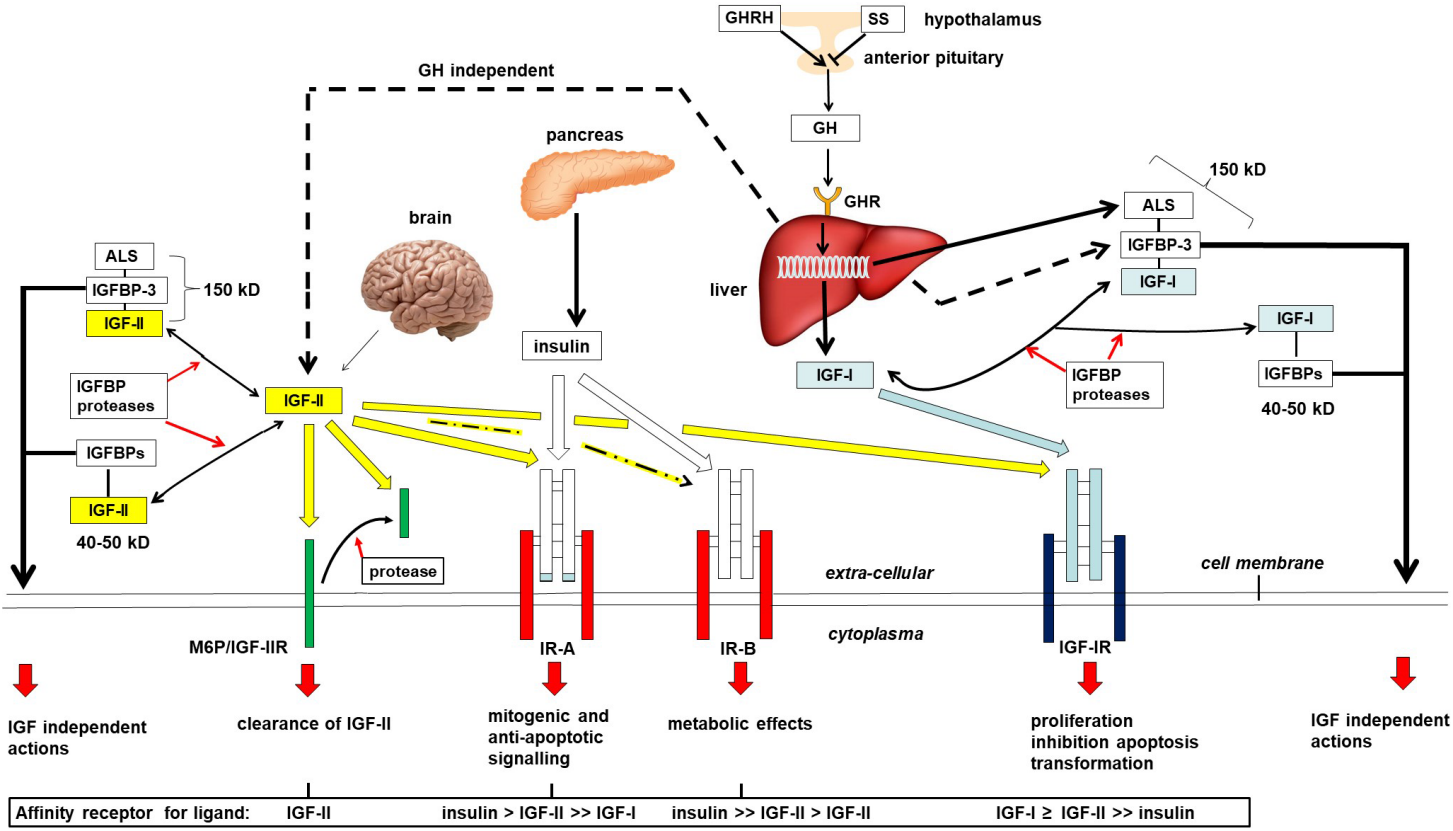
The secretion of growth hormone (GH) by the anterior pituitary gland is stimulated by hypothalamic growth hormone releasing hormone (GHRH) and inhibited by somatostatin (SS). GH activates the GHRs on the liver and various other target tissues. This may result in direct, mainly metabolic effects of GH or indirect actions such as stimulation of cell proliferation and inhibition of apoptosis being mediated by insulin‐like growth factor (IGF)‐I. IGF‐II is not directly under the influence of GH. IGFs exert negative feedback on GHRH and GH secretion and positive feedback on SS release. IGF‐I and ‐II can act in either an autocrine, paracrine, or endocrine fashion through high affinity binding to the IGF‐I receptor (IGF‐IR) which is expressed by nearly all cell types. The interaction between insulin and the IGF‐IR is relatively weak. Insulin and IGF‐II have a high affinity for the insulin receptor (IR)‐A. IR‐B exhibits high affinity binding of insulin, whereas IGF‐II is bound with a relatively low affinity. Only IGF‐II binds firmly to the M6P/IGF‐IIR. A soluble form of the M6P/IGF‐IIR, shed from the plasma membrane by proteases can be found in the circulation. When cells express both the IGF‐1R and IR, heterodimeric hybrid receptors may form which may bind both IGF‐I and IGF‐II (not shown). The liver makes an important contribution to both IGFs in the circulation where they are strongly bound to IGF‐binding proteins (IGFBPs). Stable ternary 150 kD complexes are formed by an IGF, liver derived (GH independent) IGFBP‐3 (to a lesser extent also with IGFBP‐5), and acid labile subunit (ALS) that cannot pass the blood capillaries. ALS synthesis and release by the liver is stimulated by GH. In addition, binary 40–50 kD complexes are formed consisting of an IGF and an IGFBP exhibiting a relatively shorter half‐life in the circulation. Only a very small fraction of the IGFs are present in the free, unbound form. At the tissue level, IGFs can be released from the IGFBPs by the action of various IGFBP proteases

The glucose‐lowering potential of the circulating pool of IGFs is estimated to be approximately 10 times lower than that of insulin, due to their relatively low affinities for the IR isoform B. However, on average, the total concentration of IGFs in the blood is about 1,000 times higher than that of insulin.^3^ The reason that healthy persons are not persistently hypoglycemic is that IGFs are firmly bound to protective IGFBPs in the circulation. Six different types of IGFBPs exist, designated IGFBP‐1 to ‐6.^8^, ^9^, ^10^ Besides their mainly inhibitory effect on IGF actions, the IGFBPs also exhibit IGF‐independent, intrinsic biological activities.^9^, ^10^, ^11^ The various binding proteins have different affinities for IGF‐I and IGF‐II, IGFBP‐2 and IGFBP‐6 preferentially interacting with IGF‐II. Both IGFs bind in particular to the most abundantly present IGFBP‐3, with almost equal affinity. Under normal circumstances, most (>75%) of the IGFs in the circulation are sequestered in large 150 kD ternary complexes with either IGFBP‐3 or (to a lesser extent) IGFBP‐5 and an acid labile subunit (ALS) with a serum half‐life of 12–15 hr.^3^, ^8^ ALS is produced mainly by the liver, its expression being stimulated by GH.^12^ The formation of these large complexes limits rapid exchange of IGFs between the vascular pool and the tissue compartments, and in this way contributes to the homeostasis of IGF‐bioactivity. The remainder of IGFs circulate in smaller (40–50 kD) binary complexes with IGFBP‐3 or other IGFBPs (half‐life 20–30 min). The smaller complexes have a greater capillary permeability and thus are thought to increase IGF bioavailability to the tissues. Normally, only ≤1% of the IGFs is unbound with a short half‐life in the circulation of 10–12 min.^3^ For IGFs to exert their biological effects they must be liberated from the high‐affinity IGFBPs by IGFBP cleaving proteases. Using protein sequencing and specific protease inhibitors, several proteases have been identified that are capable of cleaving IGFBP‐3 and several other IGFBPs, such as the matrix metallopeptidase pregnancy‐associated plasma protein A2 (PAPP‐A2).^13^, ^14^ Presumably, this results in destabilization of the 150 kD ternary complex and an increased bioavailability of IGFs to target tissues. The most important parts of the GH‐IGF system, including the receptors involved, are shown in Figure 1.

### 2.2 IGF‐II signaling

Most of the biological actions of both IGF‐I and ‐II (either endocrine, paracrine, or autocrine) are mediated through to the IGF‐IR. However, IGFs can also interact with the IR. The IGF‐IR and IR are structurally homologous. Both receptors are members of the subclass II of the tyrosine kinase receptor super‐family and are expressed at the cellular surface as disulfide‐linked dimers in an α2β2 configuration.^15^ The IGF‐R and IR share various intracellular signal transduction intermediates.

IGF‐I and ‐II bind to the IGF‐IR with a comparable high affinity, inducing auto phosphorylation of the β‐subunit on several tyrosine residues in the β kinase domain.^16^ This enables several adaptor proteins, including insulin response substrates, to bind, followed by activation of the phosphatidylinositol‐3‐kinase (P13K)‐protein kinase B (PKB) pathway and the mitogen‐activated protein kinase (MAPK) pathway. IGF‐IR signaling can induce cell differentiation, malignant transformation, inhibition of apoptosis, as well as an increase in glucose uptake by promoting glucose transporter 4 (GLUT4) expression at the cell surface in muscle and adipose tissue.^5^, ^16^, ^17^, ^18^

Alternative splicing of the IR gene yields two isoforms, IR‐A and IR‐B. IR‐B differs from IR‐A by the inclusion of exon 11 which encodes a 12‐amino acid sequence at the C‐terminus of the IR α‐subunit.^19^ Most cell types express both IR‐A and IR‐B but the composition differs among tissues. The IR isoforms show different functional features and it has been suggested that both isoforms have somewhat different signaling pathways. Both isoforms exhibit similar, high affinities for insulin and a very low affinity for IGF‐I.^18^, ^20^, ^21^ However, IR‐A, being expressed especially in fetal tissue and some tumor cells, also binds IGF‐II with significant affinity (~15% when compared to insulin).^19^, ^20^ It is thought that IR‐A transmits predominantly mitogenic and anti‐apoptotic signals in response to IGF‐II.^17^, ^18^, ^19^, ^22^ IR‐B is mainly expressed in muscle, liver, and adipose tissue and mediates predominantly the metabolic effects of insulin.^6^, ^18^, ^23^ Low affinity binding of IGF‐II (~2% when compared to insulin) also tends to result in insulin‐like metabolic effects. When cells express both the IGF‐1R and IR, heterodimeric hybrid receptors may form that comprise one α‐ and one β‐subunit of each receptor. Both IGF‐II and IGF‐I can bind to these hybrid receptors, especially to hybrid IGF‐IR/IR‐A, leading to effects that are closer to those of IGF‐IR.^17^, ^18^, ^22^ The biological relevance of these hybrid receptors is unclear.

The IGF‐II receptor, identical to the M6P receptor, abbreviated M6P/IGF‐IIR, is a multifunctional transmembrane glycoprotein and consists of a large extracellular part with multiple domains of which one binds IGF‐II and two others bind M6P. The exact binding interactions between the receptor and its ligands are not known.^5^, ^24^, ^25^ The M6P/IGF‐IIR has a small transmembrane unit and a short tail residing in the cytoplasm that lacks tyrosine kinase activity and an auto‐phosphorylation site. It is present in the plasma cell membrane of most cell types but also cycles between the trans‐Golgi network and endosomes. M6P/IGF‐IIR has a higher affinity for mature 7.5 kD IGF‐II than the IGF‐IR and does not bind IGF‐I and insulin. The M6P/IGFIIR gene is paternally imprinted in mice but this is still controversial in humans. M6P/IGF‐IIR was initially reported to be expressed from both parental alleles.^26^, ^27^ However, subsequent studies showed evidence that M6P/IGF‐IIR is a polymorphic trait in humans and that M6P/IGF‐IIR is imprinted during early development in 25–50% and 50% of fetuses, and first trimester placental tissues, respectively.^28^, ^29^, ^30^ Binding of IGF‐II to the M6P/IGF‐IIR at the cell surface results in endocytosis, delivery to the Golgi apparatus, and subsequent degradation of the ligand in the lysosome.^5^, ^24^, ^25^ This reduces the availability of IGF‐II for binding to the IGF‐IR. A soluble form of the M6P/IGF‐IIR, shed from the plasma membrane by proteases, that lacks the carboxyl terminal cytoplasmic and transmembrane domains, can be found in the circulation.^31^ It is not clear to what extent the soluble M6P/IGF‐IIR competes for binding to IGF‐II with the IGF‐IR, IR‐A, and the IGFBPs. Genetic evidence clearly supports a role for the M6P/IGF‐IIR in organ development and growth, and that it may play an important role in tumor progression.^24^ In addition, the M6P/IGF‐IIR is involved in the targeting of various M6P‐tagged proteins, including lysosomal proteins, from either the cell surface or the trans‐Golgi network to late endosomes via clathrin coated vesicles, and, finally, to the lysosomes. Several lysosomal storage diseases, such as Fabry and Pompe disease, are currently treated by i.v. infusion of the particular lysosomal enzyme (i.e., as M6P‐tagged recombinant protein) that is deficient or absent.^32^ The recombinant protein enters the target cells through the M6P/IGF‐IIR receptor on the cytoplasm membrane. Mutually exclusive binding of IGF‐II and naturally occurring phosphomannosyl ligands to distinct but proximal sites on the receptor suggests that the M6P/IGF‐II receptor cannot simultaneously fulfill the functional requirements of both IGF‐II and lysosomal enzymes. It is not known whether this kind of enzyme replacement therapy interferes significantly with the binding and uptake of IGF‐II at the cell surface and, as a possible consequence, increases the risk of cancer.

Although It is assumed that the M6P/IGF‐IIR functions mainly to clear IGF‐II from the cell surface to attenuate its signaling through IGF‐IR or IR, there is some evidence that IGF‐II stimulates the extracellular signal‐regulated kinase (ERK)1/2 cascade in human embryonic kidney (HEK) 293 cells via the IGF‐II receptor by triggering the sphingosine kinase‐dependent “transactivation” of a G protein‐coupled sphingosine‐1‐phosphate receptor that targets several intracellular signaling pathways.^33^

### 2.3 The IGF‐II gene and IGF‐II mRNAs

**Figure 2:**
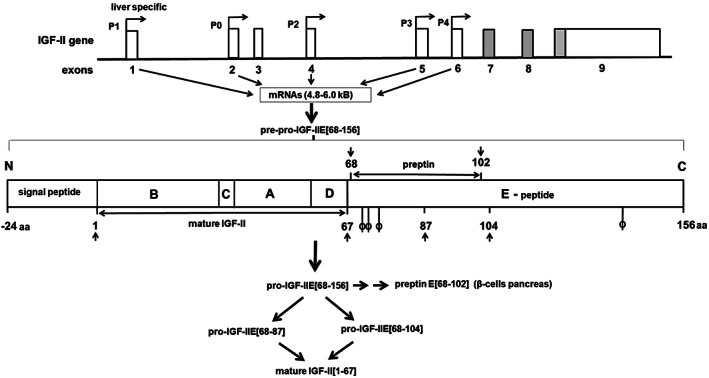
Schematic illustration of the structure of the insulin‐like growth factor (IGF)‐II gene, located on chromosome 11p15.5 with its five putative promotors, leading to mRNAs ranging between 4.8 and 6.0 kB. Only exons 7, 8, and a part of 9 encode (depicted in gray) for the pre‐pro‐IGF‐II translation product of 180 amino acids that is organized into five structural domains (A–E) and contains 3 intra‐molecular disulfide bonds between Ser residues 9 and 47, 21 and 60, and 46 and 51, respectively.^5^, ^36^ The A and B domains of mature IGF‐II are structurally analogous to mature IGF‐I and insulin. The flexible C domain is similar to the C domain in pro‐insulin but is not proteolytically cleaved, while the D domain is unique to IGF‐II. The N‐terminal signal peptide of 24 amino acids is enzymatically removed and the remaining pro‐IGF‐IIE [68–156] glycosylated, followed by sequential proteolysis (indicated by arrows) yielding mature IGF‐II^1^, ^67^ Demonstrated glycosylation sites are indicated by ϕ. Relatively stable, incompletely processed heterogeneously glycosylated or non‐glycosylated pro‐IGF‐II peptides that contain all or part of the E‐domain are also secreted, normally making up 10–20% of the total amount of circulating IGF‐II. In addition, in granules of pancreatic β cells the E‐domain derived peptide preptin [68–102] has been detected

The IGF‐II gene is located, adjacent to the insulin gene, on chromosome 11p15.5, a region which contains several imprinted genes, including two adjacent tumor suppressor genes, H19 and p57kip2. The IGF‐II gene is maternally imprinted, thus with expression resulting favorably from the paternally inherited allele.^18^, ^34^, ^35^ This genetic restraint is lost in many cancers, resulting in upregulation of both IGF‐II mRNA and protein expression, which contributes to tumorigenesis. Contrary to IGF‐II, H19, and p57kip2 are expressed from the maternal allele. Both H19 and p57kip2 play a role in conserving imprinting of the IGF‐II gene.^34^, ^35^ Normally, in several human brain regions (choroid plexus, leptomeninges, and brain endothelial cells) and in the adult liver a loss of imprinting occurs, resulting in both IGF‐II and H19 being transcribed from both parental alleles. The IGF‐II gene consists of four promotors (P1–P4) and nine exons (Figure 2). Promotor usage seems tissue specific and developmentally regulated.^17^, ^18^, ^37^ Especially during embryogenesis transcription occurs through usage of P2–P4, which seems to result always in the expression of only the paternal allele. More recently, Monk et al. found an additional promotor, P0, in front of exon 2.^38^ The human fetal IGF‐II‐P0 gene transcript is paternally expressed at high levels in fetal skeletal muscle and, later, at a lower transcribed level, it is found ubiquitously in adult tissues and term placenta.^38^ In adults, the choroid plexus, leptomeninges and, in particular, the liver exhibit biallelic expression of the IGF‐II gene by usage of P1.^17^, ^18^, ^37^ The latter phenomenon may explain the maintenance of relatively high levels of IGF‐II in the circulation of humans.

### 2.4 Posttranscriptional processing of the IGF‐II precursor protein

The first six exons of the IGF‐II gene can be transcribed into various RNAs (4.8–6.0 kB) from the different promoters, but do not code for protein. Exons 7, 8, and 9 code for the same precursor protein of 20 kD called pre‐pro‐IGF‐II. This 180‐amino acid protein can be divided into five domains (A–E) and include a N‐terminal signal peptide of 24 amino acid residues, the 67 amino acid long mature protein (with three intra‐molecular disulfide bonds), and an 89 residues extension at the COOH terminus, designated as the E‐domain.^3^, ^18^, ^34^, ^39^, ^40^ Studies on HEK 293 cells indicated that posttranslational processing of pre‐pro‐IGF‐II involves removal of the N‐terminal leader sequence and the addition of O‐linked *N*‐acetyl galactosamine residues to Ser71, Thr72, Thr75, and Thr139 in the cis‐Golgi apparatus.^41^ When pro‐IGF‐II reaches the trans‐Golgi network sialic acid is added to these sugar residues Subsequently, sequential proteolytic cleavage of the glycosylated E‐domain at Arg residues 104, 87, and 67, respectively takes place by prohormone convertases (PCs), finally leading to the mature protein^3^, ^18^, ^39^, ^40^ (Figure 2). To date, the identity of the specific PC(s) and glycosidases responsible for the physiological maturation of pro‐IGF‐II have not yet been fully clarified. There is evidence that pro‐protein convertase subtilisin/kexin type 4 (PCSK4) functions as a critical pro‐IGF‐II convertase at Arg104, at least in human placental development and pro‐IGF‐II overexpressing tumors.^42^, ^43^, ^44^ It has been demonstrated that glucose‐regulated protein (GRP) 94 associates with pro‐IGF‐II intermediates in cells that express the IGF‐II gene endogenously, as well as in transfected cells. The chaperone activity of GRP94 seems necessary for the appropriate processing of pro‐IGF‐II and the secretion of mature IGF‐II. In the absence of GRP94 activity, IGF‐II intermediates may undergo proteasome‐dependent degradation.^45^

Apparently, the processing within the E‐domain of pro‐IGF‐II is not necessarily coupled to secretion Although, under normal circumstances, the 67‐amino acid mature IGF‐II protein (largely derived from the liver) makes up by far most of the total IGF‐II in the blood, stable intermediates of incomplete prohormone processing with parts of the E‐domain still attached to the mature protein have identified and purified from human serum. The molecular weight (MW) of these preparations varied between 10 and 15 kDa, which is in part due to variably sialated O‐glycosylation on Thr 75. This high MW IGF‐II, commonly designated as “big” IGF‐II or IGF‐IIE, accounts for about 10–15% of the total IGF‐II in the serum of healthy adults.^46^, ^47^ However, in non‐diabetic obese subjects big IGF‐II levels in serum may be reduced whereas those of both free and total IGF‐II are increased when compared to values derived from matched normal weight controls.^48^ After the obese subjects had followed a 20‐week low calorie diet, leading to significant loss of weight, the concentrations of both free IGF‐II and total IGF‐II in their serum had decreased whereas that of big IGF‐II remained unchanged. Decreased serum levels of big IGF‐II were also reported by Tasca et al. who studied overweight women.^49^ The physiological meaning of these observations remains unclear but suggest a different relationship between nutritional status and big IGF‐II, and mature IGF‐II, respectively. In addition to the circulation, high MW forms of IGF‐II have also been found in the brain, cerebrospinal, amniotic, and seminal fluids, tumor cyst fluid, and in the conditioned medium of several cell types.^47^, ^50^, ^51^, ^52^, ^53^, ^54^

## 3 ROLE OF IGF‐II PRECURSOR FORMS IN NON‐ISLET CELL TUMOR INDUCED HYPOGLYCEMIA

### 3.1 Clinical and biological characteristics of NICTH

Many tumors highly express the IGF‐II gene compared to normal tissue. Over‐expression of IGF‐II mRNA in tumors has been attributed to loss of imprinting and mutations in tumor suppressor genes, possibly including loss of heterozygosity (LOH) at the M6P/IGF‐IIR locus.^5^, ^55^ In IGF‐II over‐expressing tumors the subsequent processing of the pro‐peptide may be disturbed, leading to the accumulation of high MW forms of IGF‐II, part of which is secreted into the circulation. It seems likely that many neoplastic cells do not express the pro‐IGF‐II processing machinery sufficiently to handle the relatively high amounts of pro‐IGF‐II produced. Tani et al. investigated a patient with non‐islet cell tumor induced hypoglycemia (NICTH) and a pleural solitary fibrous tumor.^43^ Their data suggest that defective PCSK4 expression in the tumor was responsible, at least in part, for impaired processing of the IGF‐II precursor. Similarly, a study by Kawai et al., on a group of patients with a solitary fibrous tumor, showed that imbalanced expression of IGF‐II and PCSK4 in tumor tissue was associated with elevated circulating levels of big IGF‐II and the syndrome of NICTH.^44^ NICTH represents an extreme case of excessive production of high MW IGF‐II, often by a large solid tumor, leading to an enormous insulin‐like activity in the body. This big IGF‐II (or IGF‐IIE) may contribute even up to 80% of the total IGF‐II in the circulation. Studying a patient with a leiomyosarcoma presenting with NICTH, Daughaday and coworkers were the first who noticed a connection with the presence of partly processed high MW precursor forms of IGF‐II in their patient's circulation, and called it big IGF‐II.^56^ Since then many case reports appeared in literature, involving a diversity of mostly large, slowly growing solid tumors of epithelial and mesenchymal origin, as summarized by for example, de Groot et al. and Dynkevich et al.^3^, ^18^, ^57^ Based on a relatively high number of cases reported so far, it seems that especially patients with a gastrointestinal stromal tumor (GIST) are at risk for developing NICTH.^58^, ^59^ The Doege–Potter syndrome, independently described for the first time by Doege and Potter in 1930, represents a specific form of NICTH secondary to a solidary fibrous tumor, the most common location being the pleural cavity, followed by the pelvis.^60^ The factual incidence of NICTH is not clear but it has been suggested that it occurs less frequently than insulinomas. However, it is questionable whether the disease is indeed so rare, as it is quite possible that many cases still remain unrecognized. This concerns especially patients with disseminated cancer, who are receiving palliative care where symptoms of neuroglycopenia are wrongly attributed to the effects of narcotics or other medications, malnourishment with or without cachexia, or critical illness, including hepatic failure.

NICTH patients typically show recurring signs of non‐ketotic hypoglycemia mostly in the (overnight) fasted state. Although autonomous symptoms of hypoglycemia may occur, neuroglycopenic features dominate such as confusion, anxiety, emotional lability, amnesia, and dizziness.^3^, ^18^, ^61^ In some cases, patients even went into a hypoglycemic coma. Presumably in roughly half of the patients with NICTH, episodes of hypoglycemia precede the discovery of tumor tissue. To combat hypoglycemia, patients may need large amounts of glucose. For example, in a NICTH patient with a differentiated adenocarcinoma of the rectum with multiple metastases to the liver we observed a glucose requirement of almost 1 kg/day.^62^ Glucose uptake studies on NICTH patients, using either 18‐fluorodeoxyglucose positron emission tomography or a glucose clamp revealed that skeletal muscle is the major site of glucose utilization.^63^, ^64^, ^65^, ^66^ Fasting serum insulin and C‐peptide levels are very low. Despite hypoglycemia, in several studies the insulin counter regulatory hormones glucagon, cortisol and GH either remained suppressed or did not increase appropriately.^3^, ^18^, ^67^, ^68^, ^69^ On the other hand, Chung and Henry reported in their paper on a patient with an intraabdominal hemangiopericytoma and NICTH that during hypoglycemia, the serum levels of glucagon and cortisol were initially elevated, but decreased when euglycemic conditions were achieved.^66^ Presumably, as a consequence of the action of big IGF‐II, lipolytic activity in adipocytes, hepatic glycogenolysis, gluconeogenesis, and ketogenesis are inhibited. Hypokalemia is also frequently observed in NICTH patients which again points to a high insulin‐like activity in the body.^61^ As expected, the insulin requirement in patients with type II diabetes may decrease substantially or even disappear after the onset of NICTH.^64^, ^70^ Although relatively rare, in addition to hypoglycemic symptoms, patients with acromegaloid soft tissue facial swelling and skin changes, such as skin tags and excessive oiliness, have been described.^3^, ^18^, ^71^ At least in these cases, these observations may point to excessive stimulation of the IGF‐IR (R).

The various treatment options for NICTH have been reviewed extensively by others.^3^, ^18^, ^61^ However, the most effective strategy still remains to remove the big IGF‐II producing tumor or reduce the tumor mass. In most cases this results at least in the normalization of glucose metabolism.

### 3.2 Involvement of big IGF‐II in the pathogenesis of NICTH

It is generally accepted that increased concentrations of big IGF‐II in the circulation and interstitial fluid are at the basis of the pathogenesis of NICTH. Obviously, this must be related to specific biochemical properties of principally naturally occurring stable variants of incompletely processed forms of pro‐IGF‐II, that is, a mix of unglycosylated and O‐glycosylated isoforms of IGF‐IIE [68–104], IGF‐IIE [68–87], and IGF‐IIE [68–156], respectively.^3^, ^72^, ^73^ However, using Western blotting with antibodies against E [68–113], we could not find evidence that the latter variant occurs in significant amounts in sera of NICTH patients.^39^ Greenall et al. found that recombinant preparations of the various O‐glycosylated IGF‐II precursors all can bind and activate the human IGF‐IR, IR‐A and IR‐B when expressed by mouse fibroblast cell lines, albeit to a different extent when compared to mature IGF‐II.^40^ A study by Marks et al. showed that recombinant unglycosylated IGF‐IIE [68–104] and mature IGF‐II stimulated signaling through both isoforms of the IR to a similar extent in mouse fibroblasts.^74^ Big IGF‐II purified from sera of NICTH patients showed a threefold higher insulin‐like activity compared to mature IGF‐II in a rat fat cell bioassay.^75^ Similarly, we noted that big IGF‐II, isolated from tumor cyst fluid from an NICTH patient with a hemangiopericytoma, stimulated IR autophosphorylation, even to a greater extent than mature IGF‐II, at high concentrations.^47^ Furthermore, we showed that recombinant unglycosylated IGF‐IIE [67–104], and natural (i.e., purified from Cohn fraction IV of human plasma) preparations of both glycosylated and enzymatically unglycosylated pro‐IGFE [67–87]^76^ were able to phosphorylate IGF‐IR of MC7 cells to a similar extent, but with a roughly two‐fold lower potency than mature IGF‐II.^77^ Rikhof et al. reported that big IGF‐II, secreted in high amounts by two cell lines derived from GISTs, acts as an autocrine survival pathway by signaling through IR‐A.^54^ In view of all these findings, it is likely that, in principle, big IGF‐II is capable to exert both mitogenic and insulin‐like effects.

Another important aspect is that there are several indications, from different directions, that the bioavailability of big IGF‐II at the tissue level must be increased. In this respect, a key finding is that in sera of NICTH patients most of the IGFs, including big IGF‐II, are not sequestered in 150 kD complexes with IGFBP‐3 and ALS, but instead predominantly circulate in binary complexes with IGFBPs, especially IGFBP‐2.^3^, ^18^, ^77^, ^78^ This is partly caused by the usually strongly reduced levels of the GH dependent ALS in NICTH sera. Since ALS has a protective role on IGFBP‐3, presumably by preventing its degradation by the action of proteases, serum IGFBP‐3 levels are decreased as well.^79^ Both tumor‐derived and recombinant preparations of various IGF‐IIE isoforms seem to bind to IGFBP‐2, IGFBP‐3, and IGFBP‐5 with an affinity similar to that of mature IGF‐II.^40^, ^80^ Besides a shortage of ALS and IGFBP‐3, there is evidence that incorporation of big IGF‐II into 150 kD ternary complex formation is severely impeded. Using an europium–labeled ALS based assay, Greenall et al. showed that recombinant glycosylated IGF‐II precursor forms, especially IGF‐IIE [67–87], showed significant (>65%) impairment of complex formation with ALS and IGFBP‐3 or IGFBP‐5.^40^ In contrast, unglycosylated IGF‐IIE [68–104] was still capable of forming a 150 kD complex with ALS and IGFBP‐3 to the same extent as mature IGF‐II, being in agreement with our own findings.^40^, ^77^ It has been postulated that the N‐linked carbohydrate chain of IGFBP‐3 interacts with epitopes in the E‐domain of glycosylated IGF‐IIE isoforms in such a way that it sterically hinders the binding of ALS to the IGF‐IIE‐IGFBP‐3 complex.^18^, ^80^ Alternatively, negatively charged sialic acid residues on O‐linked carbohydrates in the E‐domain may interact with positively charged amino acids in the binding ALS motifs of IGFBP‐3 and IGFBP‐5.^40^ However, using a different approach, we could not confirm the conclusions by Greenall et al. Addition of either glycosylated or enzymatically unglycosylated IGF‐IIE [67–87] purified from human serum, did not induce a shift of IGFBP‐3 from 150 kD toward smaller binary complexes, as revealed by size exclusion chromatography.^77^ In contrast, these IGF‐IIE preparations eluted as the free unbound form. Similarly, Elmlinger et al. found that soft tissue sarcoma cell lines secreted mainly non‐ complexed big (10–18 kD) IGF‐II.^81^ The underlying cause of these apparent different results is not yet clear. Apart from this, the presence of circulating big IGF‐II mainly in either binary complexes or in the unbound free form, implies that it passes the capillary wall much more readily. As a consequence, this gives rise to a great bioavailability of big IGF‐II for binding to the IGF‐IR and IRs. In their in vitro studies with recombinant isoforms of IGF‐IIE, Greenall et al. found that specifically glycosylated IGF‐IIE [67–87] bound M6P/IGF‐IIR poorly when compared to mature IGF‐II.^40^ This suggests that scavenging of this constituent of big IGF‐II is impaired, ensuring a further increase in its bioavailability.

Lastly, IGFBP‐2, the major IGFBP in the circulation of NICTH patients, has been shown to interact with the extra‐cellular matrix when bound to IGF‐II.^82^, ^83^ This could represent a mechanism whereby (big) IGF‐II is concentrated near its target cells. It is not clear whether the anti‐diabetic effects of IGFBP‐2 contribute to NICTH.^84^

### 3.3 Possible cascades leading to NICTH

**Figure 3:**
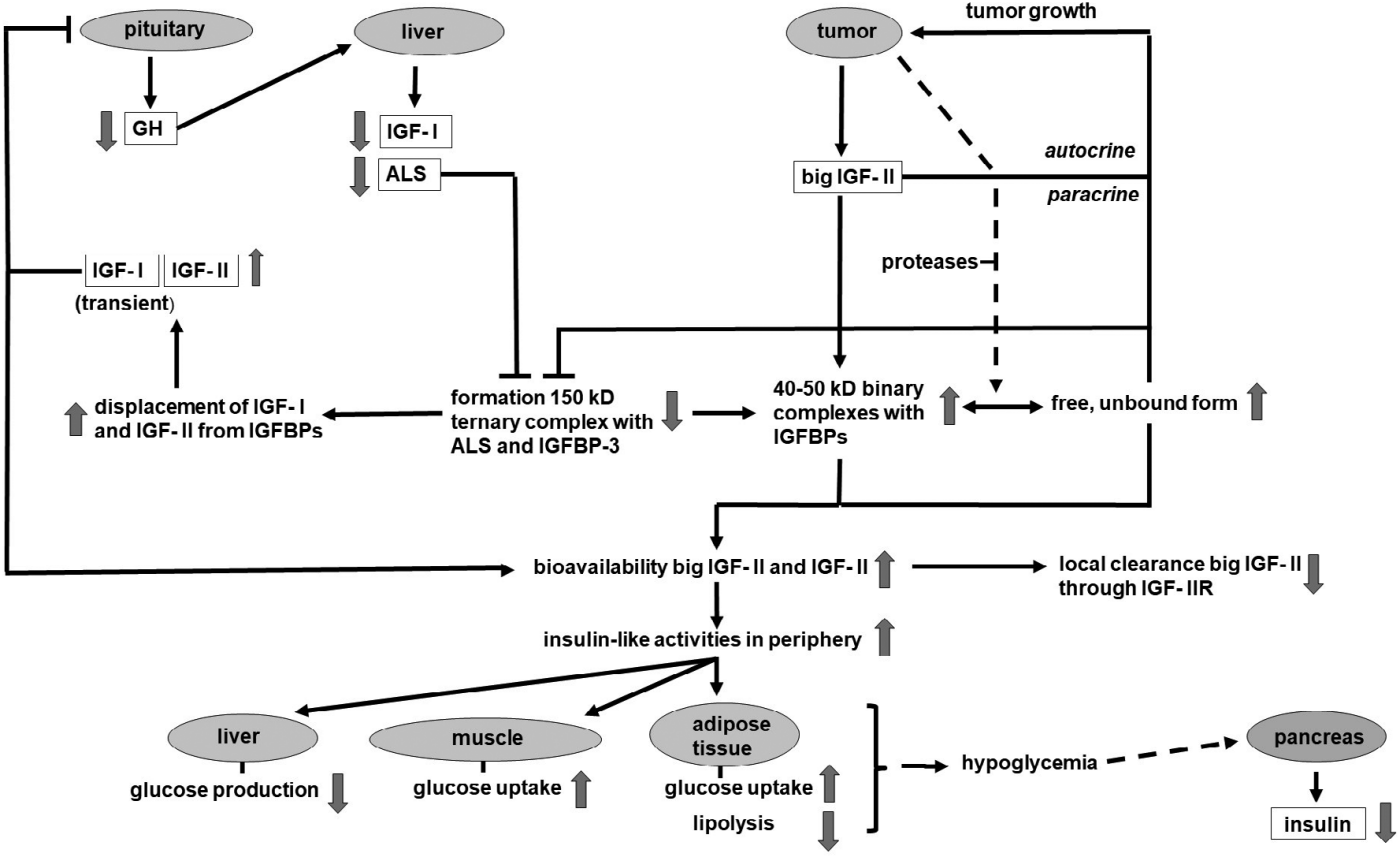
Due to insulin‐like growth factor (IGF‐II) overexpression and incomplete processing of pro‐IGF‐II increasing amounts of big IGF‐II are entering the circulation. Big IGF‐II competes with IGF‐I and IGF‐II for binding to IGF‐binding proteins (IGFBPs), which increases the concentration of unbound free IGFs. The increments of free‐IGF‐I and, possibly, also free‐IGF‐II and big IGF‐II, lead to negative feedback inhibition of pituitary growth hormone (GH) secretion followed by a decreased hepatic production of GH dependent IGF‐I and acid labile subunit (ALS). A viscous circle is set in motion whereby negative feedback on GH production (and hence ALS production) and possible inhibitory effects of big IGF‐II on 150 kD complex formation causes increasing amounts of especially big IGF‐II, but also mature IGF‐II to end up in binary complexes with IGFBPs or remain in the free form. The binary complexes and free IGFs can pass the capillary membrane relatively easy when compared with the 150 kD ternary complex. The result is that an ever‐increasing amount of (big) IGF‐II reaches the interstitial space of tissues. In contrast to mature IGF‐II, several IGF‐IIE isoforms may not effectively sequestered and internalized by the M6P/IGF‐IIR. Tumor tissue usually produces a relatively large amount of proteases that are able to liberate IGFs from inhibitory IGFBPs. Finally circulating levels of big IGF‐II and IGF‐II become sufficiently high to bind and activate the IR‐B on several target tissues to a significant extent, leading to hypoglycemia

Based on the considerations described above the following most plausible cascade of events in NICTH can be hypothesized (Figure 3). Due to various possible causes (vide infra) tumor tissue shows an increased expression of IGF‐II mRNA and its translation into protein. Depending on the type of malignant cells and the extent of IGF‐II overexpression increasing amounts of big IGF‐II are formed due to a lack or insufficiency of enzymes involved in the processing of pro‐IGF‐II. Part of the big IGF‐II enters the circulation. Initially this mainly leads to the displacement of both IGF‐II and IGF‐I from the ternary 150 kD and binary complexes, which increases the concentration of unbound free IGFs. This view is supported by Frystyk et al. who reported increased steady state serum levels of both free IGF‐I (but not total IGF‐I) and IGF‐II in a cohort of NICTH patients, as determined by an ultra‐filtration based technique and monoclonal antibody based time‐resolved immunofluorometric assays^85^. However, the molecular form of the free‐IGF‐II immunoreactivity was not determined. A similar finding was reported earlier by Daughday et al.^86^ The increments of free‐IGF‐I and, possibly, also free‐IGF‐II and big IGF‐II, lead to negative feedback inhibition of pituitary GH secretion followed by a decreased hepatic production of GH dependent IGF‐I and ALS.^87^ IGFBP‐3 in the circulation decreases concurrently because it is becoming less and less stabilized within 150 kD complexes. In fact, a viscous circle is set in motion whereby negative feedback on GH production (and hence ALS production) and possible inhibitory effects of big IGF‐II on 150 kD complex formation causes increasing amounts of especially big IGF‐II and mature IGF‐II to end up in binary complexes with mainly IGFBP‐2, IGFBP‐4, and IGFBP‐6, and/or remain in the free form. The result is that an increasing amount of (big) IGF‐II reaches the interstitial space of tissues. In addition, in contrast to mature IGF‐II, several IGF‐IIE isoforms may not effectively sequestered and internalized by the M6P/IGF‐IIR. Tumor tissue usually produces a relatively large amount of proteases that are able to liberate IGFs from inhibitory IGFBPs. There, the local level of big IGF‐II and IGF‐II becomes sufficiently high to bind and activate the IGF‐IR, IR‐A, and IR‐B to a significant extent, but is not yet high enough to reach peripheral tissues in sufficient quantities to produce effects there. Hence, initially, local mitogenic effects will dominate and stimulate tumor growth. It is likely that when the tumor grows the secretory capacity of big IGF‐II also increases further. This is illustrated by the results of a study on a cohort of patients with a GIST, showing that the patients with a relatively high tumor load (i.e., total diameter >12 cm) exhibited significantly higher pretreatment plasma levels of IGF‐IIE [68–87] than subjects with a lower tumor load.^59^ Presumably, when a certain threshold level of big IGF‐II in the circulation is exceeded, the low affinity signaling via the IR‐B becomes increasingly important, which in particular causes insulin‐like metabolic changes. This would also explain why NICTH is associated mainly with large tumors. Episodes of hypoglycemia occur when counter‐regulatory mechanisms (GH secretion is already suppressed) cannot compensate anymore for the increasing insulin‐like effects.

Although both IGF‐II and various forms of big IGF‐II bind with high affinity to the IGF‐IR and, to a lesser extent, with IR‐A in vitro, by far the majority of the numerous cases of NICTH only report the enhanced insulin like activity as the most prominent feature. Proliferative effects of big IGF‐II and IGF‐II, if any, seem to be restricted locally to tumor tissue at most. Acromegaloid features, as commonly seen in patients with elevated IGF‐I levels, are often lacking. An explanation could be that patients with NICTH are in fact GH and IGF‐I deficient which may compensate for any excessive proliferative activities of big IGF‐II and IGF‐II. This assumption is supported by the general finding that growth parameters correlate much better with changes in serum IGF‐I levels than those of IGF‐II, in spite of the in vitro finding that IGF‐I and IGF‐II both bind with high affinity to IGF‐IR and elicit the proliferation of many cell types.^8^, ^88^ On the other hand, Begemann et al. have reported a paternally inherited IGF‐II nonsense mutation in four patients from one family, associated with low birth weight and length as well as postnatal growth restriction with Silver‐Russell features.^89^ However, postnatal growth of these patients might have been affected by aberrant fetal programming. Moreover, IGF‐II levels in their circulation, presumably mainly derived from the liver that expresses IGF‐II also from the maternally allele, were still several fold higher than those of IGF‐I. The IGF‐II assay that was used made no distinction between mature and big IGF‐II. Hence, a direct role of IGF‐II and/or big IGF‐II on growth during postnatal life remains unclear until the apparent discrepancies between in vitro studies and in vivo observations cited above are solved.

### 3.4 Determination of high MW forms of IGF‐II in serum and tissue

The laboratory diagnosis and follow up of NICTH heavily relies on the finding of a relatively high concentration of big IGF in the patient's circulation with suppressed levels of insulin, C‐peptide, IGF‐I, and ALS.^18^, ^87^ In several case reports an enhanced ratio between (total) IGF‐II and IGF‐I immunoreactivities in serum, as determined by RIAs or ELISAs, has been used as a surrogate biomarker for NICTH. Measurement of total IGF‐II immunoreactivity alone is not a suitable marker since in NICTH serum levels can be either increased, lie within the normal range, or may be even reduced. The concentration of IGFBP‐2 in serum of NICTH patients is characteristically elevated.^18^, ^87^ It is likely that tumor tissue is not the source of this IGFBP‐2 but is attributable to unknown systemic mechanisms.^90^ In various cases also elevated levels of IGFBP‐6 in serum have been found,^47^, ^91^ a substantial part being produced by the tumor itself.^90^ Acid gel filtration using a BioGel P‐60 column is considered as a golden standard method for measuring big IGF‐II in serum or tissue extracts, as described for the first time by Daughaday et al.^56^ Alternatively, gel filtration through a Sephadex G‐50 column in 0.1 mol/L acetic acid can be used.^39^ Both methods allow a good separation between high molecular weight (MW) forms of IGF‐II and mature IGF‐II, and their subsequent quantitation. Several RIAs have been developed for the detection of IGF‐IIE, using either antibodies against the first 21 amino acids of the E‐domain (E [68–88]), E [69–84], or E [74–88].^39^, ^46^, ^52^, ^92^ However, these assays cannot distinguish between E‐peptide‐containing big IGF‐II and cleaved E‐ domain or fragments. The latter are increased in the circulation of patients with chronic renal failure.^39^, ^52^ We circumvented this problem by developing an ELISA that specifically detects big IGF‐II in acid Sep‐Pak C18 extracts of plasma by using a solid‐phase antibody to E [68–88] and a liquid‐phase monoclonal hIGF‐II antibody.^39^ Using a similar design, Espelund et al. developed a time resolved immunofluorometric assay with an E [78–88] antibody for coating and an europium labelled monoclonal rat IGF‐II for detection, respectively, for the measurement of IGF‐IIE in extracts of sera obtained by acidic size exclusion gel chromatography.^48^ Another, sensitive, technique that has been successfully applied for the analysis of big IGF‐II in serum is Western immune blotting, using either antibodies directed against epitopes on mature IGF‐II or the E‐domain of pro‐IGF‐II.^39^, ^93^ Recently, although applied to dog serum, a promising parallel reaction monitoring‐ mass spectrometer based method has been developed for the simultaneous quantification of the levels of IGFBP‐3, IGF‐I, IGF‐II, and big IGF‐II.^94^

Besides in Western immunoblotting, antibodies directed against either E [68–88] or E [113–134] have also been successfully used for detection of big IGF‐II in sections of tumor tissue from NICTH patients. These sections, being strongly positive for IGF‐II mRNA by in situ hybridization with an anti‐ sense digoxigenin‐labeled IGF‐II cRNA probe, revealed the abundant presence of both E [68–88] and E [113–134] immunoreactive peptides in the cytoplasm of tumor cells.^39^, ^58^, ^59^

## 4 IGF‐IIE IN HEPATITIS C‐ASSOCIATED OSTEOSCLEROSIS

Hepatitis C‐associated osteosclerosis (HCAO) represents a rare syndrome characterized by a marked increase in skeletal mass in adults who are infected with the hepatitis C virus.^95^, ^96^ Spine and hip bone mineral densities are elevated as much as twofold in these affected individuals, who represent the most dramatic example of acquired osteosclerosis in humans. Biochemical markers of bone formation are usually elevated, and transiliac bone biopsies generally show increased bone formation rates, but normal histological patterns. IGF‐I and ‐II are important skeletal growth factors that are also locally produced by osteoblasts.^97^ HCAO patients exhibit elevated serum levels of a specific precursor form of IGF‐II, that is, unglycosylated IGF‐IIE [68–104], and IGFBP‐2.^96^, ^98^ Possibly, as encountered for hepatitis‐B infections, in the liver, a hepatitis B viral protein (HBV‐X) phosphorylates the transcription factor Sp1 that can bind to promoter 4 (and probably also promoter 3) of the IGF‐II gene, leading to a considerably enhanced transcription activity and cell specific deficient processing of pro‐IGF‐II.^99^ HCAO patients have normal serum levels of IGF‐I and total IGF‐II, and somewhat reduced levels of IGFBP‐3 and ALS.^98^ In the same study it was found that the mature IGFs are largely retained in 150 kD complexes. Although IGF‐IIE [68–104] was also present as ternary complexes, it appeared to be predominantly present in more readily bioavailable 50 kD complexes, in association with IGFBP‐2. Presumably, IGF‐IIE [68–104] is secreted in sufficient molar excess to displace effectively mature IGF‐II from IGBP‐2.^82^ In vitro studies revealed a high avidity of the IGF‐II/IGFBP‐2 complex for human osteoblast extracellular matrix.^96^ Possibly, in HCAO patients, IGFBP‐2 is targeting IGF‐IIE [68–104] to the skeleton in these patients, resulting in overstimulation of bone formation. HCAO patients do not suffer from hypoglycemia,^96^ nor have NICTH patients been reported to exhibit osteosclerosis. Thus, the different clinical manifestations of IGF‐IIE and IGFBP‐2 overproduction in the two syndromes may be explained on basis of differences in biological activities and/or targeting properties between (glycosylated) IGF‐IIE [68–87] and unglycosylated IGF‐IIE [68–104], respectively, which would complement other data on this subject.^40^, ^77^ Conover et al. showed that subcutaneous administration of 7.5 kD rhIGF‐II/IGFBP‐2 complexes stimulates bone formation and prevents loss of bone mineral density in a rat model of disuse osteoporosis.^97^ It could well be that treatment with IGF‐IIE [68–104] would be much more effective than with mature IGF‐II.

## 5 PREPTIN

Preptin is a 34‐amino acid peptide hormone, first isolated in 2001 from cultured murine pancreatic β TC6‐F7‐cells, that is cosecreted with insulin, amylin, and pancreostatin from secretory granules.^100^ Pro‐IGF‐II, the precursor of preptin, has been detected in the endocrine β cells of the islets of Langerhans of both rats and humans by immune histochemical techniques.^101^ Preptin corresponds to Asp69‐Leu102 of the E‐peptide of pro‐IGF‐II, a sequence that is highly conserved between rodent and human species. It is remarkable that the N‐terminal part of the sequence of preptin starts at Asp69 and not at Arg68, and terminates at Leu102, but not at Lys104, the respective commonly nominated endoproteolytic cleavage points for PCs.^41^ This would suggest additional aminopeptidase and/or carboxypeptidase activity is involved in the IGF‐II maturation and formation of preptin. Indeed, carboxypeptidase activity in insulin‐secretory granules has been demonstrated.^102^

Several commercial ELISAs are currently available for the measurement of preptin in biological fluids. However, it is not always clear to what extent they show cross‐reactivity with IGF‐IIE isoforms.

### 5.1 Glucose homeostasis

Together with insulin, amylin, and pancreostatin, preptin is involved in glucose homeostasis. Preptin infusion into the isolated, perfused rat pancreas increases the second phase of glucose‐mediated insulin secretion by 30%, while infusion with antibodies directed against preptin decreases the first and second phases of insulin secretion by 29 and 26%, respectively.^100^ These findings suggest that preptin may be an amplifier of glucose‐mediated insulin secretion which is supported by the results of a study by. Cheng et al. who found that an intravenous preptin infusion administered to rats induced a reduction in the plasma glucose level that was related to insulin secretion during glucose loading.^103^ They also reported that the effect of preptin on insulin secretion by a mouse insulinoma cell line was similar to that of glibenclamide, which stimulates insulin secretion by inhibiting ATP‐responsive potassium channels and subsequent calcium influx in pancreatic beta cells. Addition of either an inhibitor of phospholipase C (PLC) or a phosphokinase C (PKC) inhibitor to the culture medium resulted in a concentration‐dependent decrease in insulin secretion. The effect on insulin secretion by preptin was reduced when the M6P/IGF‐IIR was blocked by antibodies. These observation led to the hypothesis that the stimulatory effect of preptin on insulin secretion acts through M6P/IGF‐IIR‐mediated stimulation of the PKC/PLC pathway. This would mean that the M6P/IGF‐IIR has also other functions than acting as a scavenger for IGF‐II and ensuring the transport of lysosomal proteins.

The possible role of preptin in glucose homeostasis has been also investigated in humans. Preptin levels appear to be elevated in the circulation of patients with metabolic disturbances including gestational diabetes mellitus, polycystic ovary syndrome, impaired glucose tolerance, type 2 diabetes mellitus, diabetic nephropathy, obesity, and overweight.^104^, ^105^, ^106^, ^107^, ^108^ Positive correlations were found between the concentration of preptin in serum and the levels of insulin, glucose, HbA1c, and the homeostatic model assessment insulin resistance (HOMA‐IR).^104^, ^105^, ^106^ These data suggest that, as with rodents, in humans preptin is cosecreted with insulin and intensifies glucose‐mediated insulin secretion. Hence, it may be useful to evaluate the therapeutic effect of preptin in patients with impaired glucose tolerance, eventually in combination with an amylin analogue, such as pramlintide, which is widely applied to improve glycemic control in diabetic patients.

### 5.2 Bone mineral metabolism

Insulin and amylin are anabolic to osteoblasts, and amylin also inhibits osteoclastic bone resorption.^109^ There is increasing evidence that the action of cosecreted preptin also contributes to the regulation of bone mass, both in rodents and humans. This is perhaps not so surprising considering the involvement of unglycosylated IGF‐IIE [68–104] in HCAO, a peptide that harbors the amino acid sequence of preptin, as discussed above. In vitro experiments by Cornish et al. yielded that preptin stimulated the proliferation of primary fetal rat osteoblasts and osteoblast‐like cell lines and decreased osteoblast apoptosis induced by serum deprivation, without affecting bone resorption.^110^ Preptin may signal osteoblast proliferation through a G protein‐coupled receptor that activates G_i_‐dependent phosphorylation of p42/44 MAPK.^110^ In the same study, local injection of preptin promoted bone formation and bone area in adult male mice. Xiao et al. found that preptin promoted the cell proliferative activity and osteoblastic differentiation in murine osteoblast‐like MC3T3‐E1 cells in a dose‐independent manner, as evidenced by an increased expression of several osteoblast‐specific genes, including alkaline phosphatase.^111^ In these cells, the Wnt/β‐catenin signaling pathway seems to be involved in the osteogenic effects of preptin. Preptin also has been shown to stimulate the proliferation and differentiation of human osteoblast cells, in this case being mediated by the ERK/MAPK/connective tissue growth factor (CTGF) pathway.^112^ There are also clinical studies that support the bone‐anabolic effect of preptin. Serum preptin and breast milk preptin were found to be lower in rachitic children and their mothers, respectively, when compared to non‐rachitic subjects.^113^ Serum preptin levels are decreased in both men and women with osteoporosis and positively correlate with bone mass density.^114^, ^115^ It is therefore likely that a lower activity of preptin is involved in the pathogenesis of osteoporosis, affecting bone formation rather than bone resorption. In contrast, elevated serum preptin levels have been found in obese and overweight subjects.^107^ It is tempting to speculate that increased levels of several nutrition‐related hormones derived from the pancreatic β‐cell (including preptin), and adipose tissue, that are observed in the peripheral circulation of overweight subjects,^109^ contribute to the higher bone mass and lower fracture risk associated with obesity.

It has been shown that the N‐terminal truncated sequence of preptin, that is, preptin [1–16] retains the full preptin‐like bone‐anabolic activity in rodents both in vitro and in vivo.^116^, ^117^ Unlike the full‐length preptin, preptin [1–16] has no effect on insulin secretion. Kowalczyk et al. synthesized several truncated analogues of preptin [1–16] and investigated their ability of to stimulate osteoblast proliferation.^116^ They identified a short fragment, preptin [1–8], that still has a stimulating effect on the proliferation of rat osteoblasts and bone nodule formation. This peptide is believed to be suitable as a leading compound for the development of orally active antiosteoporosis drugs.^116^

### 5.3 Vascular calcification

Conditions of hyperinsulinemia, as with glucose intolerance and insulin resistance, are associated with elevated serum levels of cosecreted preptin (vide infra) and are generally considered to be an important risk factor for the development of vascular calcification.^118^, ^119^ Elevated serum preptin levels as seen in obese and overweight subjects appeared to correlate inversely with those of osteocalcin, which may stimulate vascular smooth muscle mineralization and differentiation.^107^ Li et al. investigated a cohort of subjects with suspected cardiovascular disease.^119^ The serum preptin level was significantly elevated in a subgroup with coronary artery calcification (CAC) when compared to a non‐CAC subgroup. It was even proposed that serum preptin is a good independent predictor of CAC.^119^ The prevalence of glucose intolerance or insulin resistance was not investigated. All these studies point to preptin as an important intermediary for the occurrence of crosstalk between pancreatic β cells and bone. The underlying molecular mechanism by which preptin exerts its effects on vascular calcification remains to be elucidated.

## 6 CONCLUDING REMARKS

The translation product of the IGF‐II gene undergoes complex posttranslational processing to form the mature protein. Several intermediates as well as a fragment of the E domain without an IGF‐II moiety exhibit biological activities that even differ from the mature protein in several aspects. In this regard, the IGF‐II precursor protein resembles pro‐glucagon or pro‐opiomelanocortin whose cell type specific processing also results in different bioactive proteins. The excessive production and apparent high bioavailability of predominantly heterogeneously glycosylated pro‐IGF‐IIE [68–87], big IGF‐II, is causally associated with disturbance of the glucose homeostasis, that is, enhanced insulin‐like effects, in the disease picture of NICTH. Interestingly, preptin, derived from pancreatic β‐cell specific additional endo‐proteolytic processing of the E‐domain of pro‐IGF‐II, is also involved in glucose homeostasis as an amplifier of glucose‐mediated insulin secretion. In HCAO, a pro‐IGF‐IIE variant harboring a longer amino acid sequence of the E‐domain, pro‐IGF‐IIE [68–104], seems to be the major culprit. Probably the hepatitis C virus induces increased transcription of the IGF‐II gene in the liver and apparent deficient processing of pro‐IGF‐II, selectively at arginine 104, resulting in increased release of pro‐IGF‐IIE [68–104] in the circulation. The main effect of pro‐IGF‐IIE [68–104] appears to be to increase bone formation and bone mineral density, a property its shares with preptin. Besides glucose homeostasis and bone turnover, there are indications, so far at least in mice, that pro‐IGF‐II is also involved in yet another biological process, that is, recovery from muscle injury.^120^ It was shown that high molecular weight variants of IGF‐II, but not mature IGF‐II, are specifically expressed during muscle regeneration being delayed and decreased in aged animals. Supplementation with a 293 HEK cells derived recombinant 25 kD glycosylated pro‐IGF‐II preparation stimulated the regenerative response by promoting the proliferation of satellite cells, angiogenesis, and suppressing adipogenic differentiation of platelet‐derived growth factor receptor α^+^ mesenchymal progenitor cells. Further research into the latter process revealed that mature IGF‐II had no effect on this.^120^ Based on the above, it can be argued that it is worthwhile to conduct more research into the possible therapeutic values of pro‐IGF‐II variants and preptin (or derivatives thereof) in the treatment of type 2 diabetes, osteoporosis and complicated fractures, and muscle regeneration, respectively. Finally, one may question how, in general, the biological roles of pro‐IGF‐II variants and preptin relate to that of mature IGF‐II. In the various studies on biological effects of IGF‐II, one had or could not distinguish between pro‐IGF‐II variants or mature IGF‐II. It is therefore not excluded that, in fact, the locally produced pro‐IGF‐II variants are important for achieving most of the biological effects and that what we call mature IGF‐II is more or less a degradation product that is released into the circulation, firmly bound to IGFBPs and quickly sequestered via the M6P/IGF‐2 receptor which is present on nearly all cell types.
